# Evaluating the Compatibility of Spinosad and Alpha-Cypermethrin for Controlling Six Insect Pests Infesting Stored Wheat

**DOI:** 10.3390/insects14110855

**Published:** 2023-11-02

**Authors:** Waqas Wakil, Nickolas G. Kavallieratos, Nikoleta Eleftheriadou, Muhammad Asrar, Taha Yaseen, Muhammad Tahir, Khawaja G. Rasool, Mureed Husain, Abdulrahman S. Aldawood

**Affiliations:** 1 Department of Entomology, University of Agriculture, Faisalabad 38040, Pakistan; mtyh786@gmail.com; 2 Senckenberg German Entomological Institute, D-15374 Müncheberg, Germany; 3 Laboratory of Agricultural Zoology and Entomology, Department of Crop Science, Agricultural University of Athens, 75 Iera Odos Str., 11855 Athens, Greece; nikolelef@aua.gr; 4 Department of Zoology, Government College University, Faisalabad 38000, Pakistan; asrar@gcuf.edu.pk; 5 Ministry of National Food Security and Research, Islamabad 44000, Pakistan; tahir_1558@yahoo.com; 6 Department of Plant Protection, College of Food and Agriculture Sciences, King Saud University, P.O. Box 2460, Riyadh 11451, Saudi Arabia; gkhawaja@ksu.edu.sa (K.G.R.); mbukhsh@ksu.edu.sa (M.H.); aldawood@ksu.edu.sa (A.S.A.)

**Keywords:** insecticide combinations, pyrethroid, spinosyn, pest management

## Abstract

**Simple Summary:**

This study investigated the effectiveness of combining spinosad and alpha-cypermethrin against major wheat storage pests, including *Rhyzopertha dominica*, *Tribolium castaneum*, *Cryptolestes ferrugineus*, *Sitophilus oryzae*, *Oryzaephilus surinamensis*, and *Trogoderma granarium*. Spinosad was tested at two concentrations and alpha-cypermethrin was used at one concentration in laboratory conditions. The results show that combining both treatments led to higher pest mortality compared to individual treatments. The most effective treatment combination was the application of the high dose of spinosad with alpha-cypermethrin, resulting in the highest mortality and substantial progeny reduction. *Trogoderma granarium* was found to be the most tolerant pest, followed by *O. surinamensis*, *C. ferrugineus*, *T. castaneum*, *S. oryzae*, and *R. dominica*. This study suggests that combining alpha-cypermethrin and spinosad can be an effective strategy for protecting stored wheat against multiple insect pests.

**Abstract:**

The deterioration of stored wheat due to pest infestations is a significant concern, with pests like *Rhyzopertha dominica*, *Tribolium castaneum*, *Cryptolestes ferrugineus*, *Sitophilus oryzae*, *Oryzaephilus surinamensis*, and *Trogoderma granarium* being major contributors. This study examined the efficacy of spinosad and alpha-cypermethrin, individually and in combination, against these pests under laboratory conditions. Spinosad was tested at two concentrations (0.05 and 0.1 mg/kg), while alpha-cypermethrin was applied at 0.05 mg/kg. The combined application of both insecticides led to significantly higher pest mortality compared to single treatments. Importantly, all treatments caused substantial pest mortality and exhibited the ability to suppress pest progeny production over time, as observed in both laboratory and persistence trials. Among the various treatment combinations, the joint application of 0.1 mg/kg spinosad and 0.05 mg/kg alpha-cypermethrin emerged as the most effective, resulting in elevated mortality and a marked reduction in pest progeny. *Rhyzopertha dominica* exhibited the highest susceptibility among the pests, followed by *S. oryzae*, *T. castaneum*, *C. ferrugineus*, *O. surinamensis*, and *T. granarium*. The remarkable performance of the joint action of alpha-cypermethrin and spinosad at low doses highlights this combination as an efficacious approach for safeguarding stored grain against these destructive insect pests, warranting further exploration.

## 1. Introduction

Among the 1663 insect species identified as pests of stored grain worldwide [1], *Oryzaephilus surinamensis* (L.) (Coleoptera: Silvanidae), *Rhyzopertha dominica* (F.) (Coleoptera: Bostrychidae), *Cryptolestes ferrugineus* (Stephens) (Coleoptera: Laemophloeidae), *Sitophilus oryzae* (L.) (Coleoptera: Curculionidae), *Trogoderma granarium* Everts (Coleoptera: Dermestidae), and *Tribolium castaneum* (Herbst) (Coleoptera: Tenebrionidae) are some of the most significant threats for stored commodities. Wheat and cereals, among other commodities worldwide, are subjected to deterioration by these voracious feeders, causing significant grain losses and product degradation [1,2,3,4]. A significant proportion of the universal production of grains is lost annually due to pests’ damage. In developed countries, grain loss caused by insect pests amounts to nearly 9%, whereas developing nations experience losses in storage ≥20% [5,6]. The imperative need for pest control has led, however, to an excessive use of chemical pesticides, especially organophosphates and pyrethroids, as grain protectants [7]. It has now become apparent that this exaggerated application has resulted in resistance emergence for various storage pests, therefore minimizing the efficiency of pest management strategies [7,8,9,10]. A strategic approach includes the utilization of combinations of insecticides from either the same or distinct chemical groups. This combination has the potential to amplify the toxic effects of each insecticide through the generation of joint interactions. Such a blend could present an economically advantageous method of pest control, as it would require a reduced quantity of component insecticides, fewer applications over a condensed timeframe, and may involve minimal or comparatively limited risks to both farmers and the environment [11,12].

Spinosad, a bacterial-based natural insecticide, is a fusion of spinosyn D and spinosyn A, products of the actinomycete *Saccharopolyspora spinosa* Mertz & Yao (Pseudonocardiales: Pseudonocardiaceae) [13]. Spinosad, like all spinosyns, is characterized by reduced environmental and toxicological risks [14,15]. While references to in vivo toxicity in mammals have been made, it is important to emphasize that these concerns typically pertain to considerably high concentrations, with a reported range of 50% lethal concentration spanning from 0.214 to 5000 mg/kg [13]. Its mode of action involves directly targeting nicotinic acetylcholine receptors (nAChRs) in the organism’s nervous system, serving as an allosteric modulator, which leads to rapid excitation of the nervous system via stimulation of nAChRs and γ-aminobutyric acid receptors (GABA receptors), ultimately resulting in paralysis and death [13,16,17]. The effectiveness of spinosad against a range of storage insect pests, including *T. castaneum*, *C. ferrugineus*, *O. surinamensis*, *R. dominica*, *S. oryzae*, and *T. granarium*, has been well-documented in numerous laboratory and field studies, as reported in various investigations in the past, exhibiting promising results [18,19,20,21,22].

Alpha-cypermethrin, a type II pyrethroid, is a non-systemic insecticide exhibiting contact and stomach action against insects. Comprising the active isomer of the synthetic pyrethroid insecticide cypermethrin, alpha-cypermethrin demonstrates high efficacy against a broad spectrum of chewing and sucking insects, including flies, mosquitoes, and other pests found in public and animal housing [23,24,25]. Alpha-cypermethrin is considered safe for occupational and general population exposure, as well as for the environment, when used in accordance with recommended practices and application rates (0.05–1 mg/kg in crops) [26,27]. While alpha-cypermethrin has shown its effectiveness, over-reliance on it has led to the emergence of resistance among various insect pests globally, forcing users to escalate dosages, thereby increasing control costs, and yielding adverse environmental and public health consequences [28,29,30]. Several stored-grain species, including *R. dominica*, *T. granarium*, *S. oryzae*, *Tenebrio molitor* L. (Coleoptera: Tenebrionidae), *Prostephanus truncatus* (Horn) (Coleoptera: Bostrychidae), and *Tribolium confusum* Jacquelin du Val (Coleoptera: Tenebrionidae), have been subjected to alpha-cypermethrin, exhibiting varying susceptibility depending on the dose, exposure, and insect species [31,32,33,34].

Preserving grain quality is of utmost importance in any comprehensive insect pest control program, aimed at ensuring consistent quality over time. While spinosad has undergone evaluations for its efficacy against numerous stored-grain pests, the literature on the efficacy of alpha-cypermethrin is limited, while their combined effectiveness remains unexplored. Given the increasing demand for effective pest control, it is imperative to investigate this combination thoroughly. Hence, this study is designed to examine the single and combined efficacy of alpha-cypermethrin and spinosad against *T. castaneum*, *O. surinamensis*, *R. dominica*, *C. ferrugineus*, *S. oryzae*, and *T. granarium* through laboratory and persistence bioassays, aiming to explore their efficacy and practical implications for pest management strategies.

## 2. Materials and Methods

### 2.1. Insect Culture

Healthy populations of *T. castaneum*, *O. surinamensis*, *R. dominica*, *C. ferrugineus*, *S. oryzae*, and *T. granarium* were sourced from the Department of Entomology at the University of Agriculture, Faisalabad. These populations had been maintained for over a decade without exposure to any insecticide. Adult *T. granarium* specimens used in the experiment were less than 24 h old, while *O. surinamensis*, *C. ferrugineus*, *T. castaneum*, *R. dominica*, and *S. oryzae* adults were less than two weeks old. The populations of *R. dominica*, *T. granarium* and *S. oryzae* were cultured on intact wheat grains under conditions of 30 °C, 65% RH, and 24 h of complete darkness. *Oryzaephilus surinamensis* populations were reared on a mixture with a ratio of 5 parts broken wheat to 1 part brewer’s yeast and 5 parts oat flakes. Meanwhile, the populations of *T. castaneum* and *C. ferrugineus* were maintained under the same conditions but were reared on wheat flour, supplemented with 5% brewer’s yeast [35,36].

### 2.2. Grain

Before commencing the studies, the wheat employed in the bioassay, *Triticum aestivum* L. (var. Noor 2013), was prepared through sieving to ensure it is free of infestation and impurities. Subsequently, it was stored at −13 °C for 7 days to eliminate any potential insect infestation [37] and tempered at approx. 26 °C and 50% RH. A moisture content of 11.7%, was verified by a calibrated moisture meter (Dickey-John Multigrain CAC II, Dickey-John Co., Auburn, IL, USA).

### 2.3. Formulations

The spinosad formulation (Tracer 240 SC, Dow Agro Sciences, Karachi, Pakistan) containing 240 g/L active ingredient (a.i.) and alpha-cypermethrin formulation (Bestox 5 EC, FMC United Pvt. Ltd., Lahore, Pakistan) with 5% of a.i. were employed in the bioassays.

### 2.4. Laboratory Bioassays

The bioassay investigations were initiated subsequent to the preliminary experimental phase, for the evaluation of the two different concentrations of spinosad, along with the concentration of alpha-cypermethrin, against all arthropod pests. The experiment consisted of five different treatments plus a control group. The treatments involved the sole application of alpha-cypermethrin at 0.05 mg/kg wheat (AC), the sole application of spinosad at 0.05 mg/kg wheat (S1), the sole application of spinosad at 0.1 mg/kg wheat (S2), the combined application of alpha-cypermethrin at 0.05 mg/kg and spinosad at 0.05 mg/kg wheat (S1 + AC), and the combined application of alpha-cypermethrin at 0.05 mg/kg and spinosad at 0.1 mg/kg wheat (S2 + AC). Additionally, a control group was part of the experimental setup. A total of 18 lots, each containing 1 kg of wheat, were prepared for the study. Among these, 9 lots were initially treated with alpha-cypermethrin at 0.05 mg/kg wheat (AC). Within this subset of 9 lots, three lots were not further treated (AC) and six lots received an additional treatment consisting of spinosad at 0.05 mg/kg wheat (3 lots) (S1 + AC) or spinosad at 0.1 mg/kg wheat (3 lots) (S2 + AC). Of the remaining 9 lots, 6 lots were split into two groups: three lots were exclusively treated with spinosad at 0.05 mg/kg wheat (S1), and the other three lots were treated with spinosad at 0.1 mg/kg wheat (S2) [31,38]. The remaining three batches of 1 kg wheat were designated as controls. For the treatment application, each 1 kg lot was uniformly distributed in the form of a thin layer over a tray, and 1 ml of either spinosad or alpha-cypermethrin solution corresponding to each dose was applied using an airbrush (Master Multipurpose Airbrush, San Diego, CA, USA). Distinct airbrushes were employed for each aqueous solution. In the control group, the wheat underwent a similar treatment, with the same quantity of distilled water applied in the same manner using an airbrush exclusively designated for controls. Following the treatment application, the treated grains were transferred into 3-L glass jars with open mouths, which were then sealed with their lids. The jars underwent manual agitation for approximately 10 min to ensure the consistent dispersion of the insecticide solution. Subsequently, three wheat samples of 100 g were extracted from each treatment and placed inside plastic vials measuring 125 mm in height and 75 mm in diameter. The weight of the wheat samples was determined using an ELB 300 electronic balance (Shimadzu, Kyoto, Japan), with a fresh thin layer employed for each weighing session. In order to maintain sample integrity and prevent any potential cross-contamination, a distinct scoop was employed for each individual glass jar throughout the sampling procedure. In the center of the lid of each glass vial, a 15 mm diameter hole, adequately covered with gauze, was incorporated to facilitate aeration within the vial. Subsequently, 50 adult insects of mixed sex for each tested species were introduced into each glass vial. To avert the potential escape of insects, the inner neck of all glass vials was effectively sealed with polytetrafluoroethylene (Sigma-Aldrich Chemie GmbH, Taufkirchen, Germany). The vials were placed in an incubator at a controlled temperature of 30 °C and 65% RH. Mortality data were recorded at 3-, 7-, and 14-days post-exposure. This assessment was conducted by gently prodding the individuals using a brush under a Leica stereomicroscope (Wild M3B, Heerbrugg, Switzerland) to elicit movement. Separate vials were designated for every exposure interval. The offspring production by the tested adult pests was also assessed for each treatment. Therefore, after 14 days, both deceased and surviving adult insects were removed, and subsequently the vials were stored in the incubator under the same environmental conditions to monitor progeny production. The counting of progeny for *O. surinamensis* and *C. ferrugineus* was conducted 65 days later, for *T. castaneum* and *R. dominica* 62 days later, 60 days later for *S. oryzae* and 46 days for *T. granarium* [38,39,40]. For *S. oryzae* and *R. dominica*, only adults were considered for progeny determination, as the immature stages of both species remain inside the grain. Immatures and adults were recorded as progeny for the remaining species. The entire procedure was repeated a second time, involving the preparation of new wheat lots, vials, and insects for each iteration.

### 2.5. Persistence Bioassays

Following a 7-day initial exposure period of the insects to sprayed wheat, an evaluation of the treatments applied on wheat was conducted over a 120-day period by performing five bioassays at intervals of 30 days (i.e., at 0, 30, 60, 90, and 120 days) under controlled conditions of 65% RH and 30 °C, following the same procedures as in the laboratory bioassays. In this context, the term “day 0” represents the first day following the 7-day exposure period to the treated wheat. Throughout the entire duration of the experiment, three batches of 100 g of wheat, treated with the respective insecticides, were stored in sealed jars under the same temperature and RH conditions as mentioned above. For each interval, different jars were implemented. The mortality of all tested insects was assessed after 7 days of exposure to the insecticides, and their offspring was evaluated using the previously described methods. The entire process was carried out in duplicate, with each iteration involving the preparation of new wheat lots, vials, and insects.

### 2.6. Statistical Analysis

The mortality rates documented for all insect species in the control treatments were <5%. In both the laboratory and persistence trials, mortality data underwent correction using Abbott’s equation [41] and were subsequently subjected to log (x + 1) transformation to ensure normal variance before statistical analysis [42,43]. Mortality data from the laboratory trials were analyzed through a two-way analysis of variance (ANOVA), wherein the exposure interval and treatment served as the main effects, with mortality as the response variable. The analysis considered interactions among all main effects. The assessment of offspring in laboratory bioassays involved a two-way ANOVA, with treatment and insect species as the main effects, and the count of the succeeding generation individuals as the response variable. The mortality data stemming from the persistence trials were subjected to a two-way ANOVA, with the storage period and treatment as the main effects and mortality as the response variable. The assessment of offspring in persistence bioassays involved a two-way ANOVA, with treatment and storage period as the main effects, and the count of the emerging individuals as the response variable. Interactions between the main effects were also incorporated into the analysis. To make comparisons in progeny and mortality means, the Tukey–Kramer (HSD) test at a significance level of *p* = 0.05 was employed [44]. All statistical analyses were executed using Minitab, 2017 [45].

## 3. Results

### 3.1. Adult Mortality in Laboratory Bioassays

All the main effects and their interaction were significant for all six insect species tested (Table 1). For all insect species, mortality increased as the exposure interval increased (Table 2). Although no complete (100%) mortality was observed, higher mortalities were recorded in combined treatments, with the S2 + AC treatment being the most effective, followed by S2, S1, and AC. Overall, mortalities tended to be significantly higher in combined treatments compared to single applications, while differences in mortalities resulting from the S1 and the AC treatments mostly remained non-significant. Irrespective of the treatment and exposure interval, *R. dominica* was the most susceptible, followed by *S. oryzae*, *T. castaneum*, *C. ferrugineus*, *O. surinamensis*, and *T. granarium*.

For *R. dominica*, significantly high mortality was demonstrated for the combined treatments at both their highest (98.29%) and lowest dose (87.00%) at 14 days post-exposure, followed by S2 (59.05%). Single applications of spinosad at the lowest dose and alpha-cypermethrin did not yield more than 48% mortality. At 7 days post-exposure, the combined treatments exhibited significantly higher mortality compared to the single treatments (66.10% and 49.53% for S2 + AC and S1 + AC, respectively). A similar trend was observed at 3 days post-exposure, with the highest observed mortality being 43.32% for the S2 + AC treatment. The minimum mortality rates were observed for AC at three days post-exposure (14.40%). Significant differences in the mortality of *R. dominica* were observed among the exposure intervals for all treatments except for the S2 treatment.

For *S. oryzae*, a significantly high level of mortality was evident in the combined treatments, both at their maximum (93.87%) and minimum (84.62%) dose, 14 days after exposure. Subsequently, S2 also demonstrated significant mortality rates (56.97%) compared to the remaining single treatments. After 7 days of exposure, S2 + AC exhibited significant mortality (62.36%) compared to all other treatments. Similarly, at 3 days post-exposure, the highest mortality was observed for S2 + AC (40.27%). Minimum mortality was observed for AC at three days post-exposure (12.72%). Among the exposure intervals, significant differences were exhibited for all treatments apart from S1.

Regarding *T. castaneum*, significantly high mortalities were observed for S2 + AC at both 7- and 14-days post-exposure (56.59% and 89.75%, respectively). At 14 days post-exposure, significantly higher mortality rates were observed for S2 + AC, followed by S1 + AC (81.56%), and S2 (51.19%). S1 (40.60%) and AC (32.74%) did not exhibit significant differences in mortalities. At 7 days after exposure, S2 + AC exhibited significantly higher mortality rates (56.59%) compared to all other treatments that exhibited <50% mortalities. Similarly, at 3 days post-exposure, the highest mortality rates resulted from S2 + AC (37.22%), followed by all other treatments, which exhibited mortalities under 32%. Minimum mortality was observed for AC (10.04%) at 3 days after exposure. Significant differences were observed among all exposure intervals in all treatments apart from S1.

The combined treatment of S2 + AC exhibited higher mortality rates of *C. ferrugineus* compared to the remaining treatments at all exposure intervals. After 14 days of exposure, *C. ferrugineus* exhibited significantly higher mortality for S2 + AC (82.24%), followed by S1 + AC (77.45%), and S2 (46.76%). S1 and AC exhibited the lowest mortalities (36.18% and 27.30%, respectively), demonstrating non-significant differences between them. After 7 days of exposure, S2 + AC exhibited significantly higher mortality (51.51%), followed by S1 + AC (36.27%) compared to all other treatments, which resulted in less than 32% mortalities. Significant differences were exhibited in mortalities among all exposure intervals for all treatments. 

For *O. surinamensis*, the S2 + AC exhibited higher mortality rates compared to all other treatments at all exposure intervals. The highest mortality rates were observed for S2 + AC and S1 + AC (76.80% and 67.56%, respectively) after 14 days of exposure, followed by significantly lower mortalities resulting from S2 (41.66%), S1 (32.38%), and AC (32.38%). At 7 days of exposure, S2 + AC resulted in the significantly highest mortality of 47.15%, followed by S1 + AC (32.87%), while all other treatments resulted in less than 28% mortalities. After a 3-day exposure period, S2 + AC and S1 + AC resulted in 29.52% and 24.17% mortalities, respectively, exhibiting significant differences from the remaining treatments, for which mortalities remained under 17%. Mortality differences were significant across all exposure periods for each treatment.

*Trogoderma granarium* demonstrated significantly higher mortality for the combined treatments at both 3- and 14 days post-exposure. The highest mortality was observed for S2 + AC, 14 days post-exposure (70.31%), followed by S1 + AC (62.11%), S2 (37.21%), S1 (29.66%), and AC (20.50%). After 7 days of exposure, *T. granarium* demonstrated significantly higher mortality for the S2 + AC treatment (44.42%), followed by S1 + AC (29.81%), while the remaining treatments resulted in <23% mortalities. Similarly, at 3 days after exposure, S1 + AC and S2 + AC exhibited significantly higher mortality rates (26.16% and 20.76%, respectively) compared to all other treatments, which exhibited less than 14% mortalities. Differences in mortalities were significant among all exposure intervals and treatments.

### 3.2. Progeny Production in Laboratory Bioassays

Regarding offspring production, all main effects and their interaction were significant (Table 3). The minimum progeny production was recorded for S2 + AC, although not suppressed, followed by S1 + AC, S2, S1, and AC among all insect species (Table 4). The combined treatments resulted in significantly lower offspring production compared to single ones. Among all insect species, *R. dominica* exhibited the lowest progeny for S2 + AC (7.98) and S1 + AC (19.53), while the single treatments resulted in more than 46 offspring individuals. Following *R. dominica*, *S. oryzae* exhibited a progeny of 15.96 and 24.76 individuals, and *T. castaneum* 24.8 and 31.51 individuals for the respective treatments. Progeny was less affected for *C. ferrugineus* with S2 + AC resulting to 31.5, and S1 + AC to 37.73 individuals, followed by *O. surinamensis* with S2 + AC resulting to 40.1, and S1 + AC to 65.93 individuals, and *T. granarium* with 58.08 and 45.75 individuals for the respective treatments. 

### 3.3. Adult Mortality in Persistence Bioassays

After a 7-day exposure period, all the main effects were significant for all tested species. The interaction of treatment × storage period was significant only for *C. ferrugineus*, *O. surinamensis*, and *T. granarium* (Table 5). Although no complete mortality was observed, S2 + AC was the most effective treatment among all treatments and for all insect species, followed by S1 + AC, S2, S1, and AC (Table 6). For all insect species, mortality decreased over time, while combined treatments resulted in significantly higher mortalities compared to single treatments. *Rhyzopertha dominica* was most susceptible regardless of the treatment, followed by *S. oryzae*, *T. castaneum*, *C. ferrugineus*, *O. surinamensis*, and *T. granarium.*

The mortality rate of *R. dominica* reached the maximum mortality of 94.52% in wheat treated with S2 + AC at the initiation of the experiment (day 0), followed by S1 + AC (82.91%), and S2 (53.54%). The remaining treatments resulted in less than 35% mortality. After a 120-day storage period, *R. dominica* exhibited the highest mortality when treated with S2 + AC (69.28%), followed by S1 + AC (50.85%), while the lowest observed mortality was 14.32% for AC. 

Regarding *S. oryzae*, the maximum mortality at day 0 was observed for S2 + AC (87.04%), followed by S1 + AC (78.52%), and S2 (50.48%). The remaining treatments resulted in <40% mortalities. At day 120 of storage, the highest mortality was observed for S2 + AC (61.76%) and S1 + AC (46.08%). The lowest mortality rate was exhibited for the application of the AC treatment (10.58%).

The highest mortality rate of *T. castaneum* at day 0 resulted from the two combined treatments of S2 + AC (80.20%) and S1 + AC (72.01%), followed by S2 at 46.4%. The remaining treatments did not result in more than 37% mortality. After 120 days, maximum mortality was exhibited for S2 + AC (57.68%), followed by S1 + AC (41.29%), while the lowest observed mortality was for AC (8.2%).

Regarding *C. ferrugineus*, at day 0 the maximum mortality was demonstrated for the combined treatments of S2 + AC (75.09%) and S1 + AC (66.75%), followed by S2 (40.59%). The S1 and AC treatments resulted in <32% mortality. At a 120-day storage period, combined treatments resulted in the highest mortalities with S2 + AC causing 52.53% and S1 + AC 37.55% mortality. The minimum mortality was demonstrated for AC at 6.48%.

Concerning *O. surinamensis*, S2 + AC and S1 + AC had a greater impact on mortality compared to single treatments exhibiting 69.26% and 61.40%, respectively, at the first day of the bioassay. S2 exhibited 36.17% mortality, while the remaining treatments did not exceed 29% mortality. After 120 days, *O. surinamensis* demonstrated the highest mortality of 46.75% when treated with S2 + AC, followed by S1 + AC (32.07%), while the lowest mortality rate was observed for AC (5.12%).

For *T. granarium*, maximum mortality was achieved with the application of S2 + AC (63.81%), with S1 + AC (56.30%) and S2 (31.75%) following. After a storage period of 120 days, maximum mortality reached 40.6% for S2 + AC, and 27.31% for S1 + AC. The minimum mortality was demonstrated for the single application of AC (3.08%).

### 3.4. Progeny Production in Persistence Bioassays

Regarding offspring production, the main effects significantly affected progeny only in *C. ferrugineus* and *T. granarium*, while the interaction of treatment × storage period was insignificant for all insect species (Table 7). The combined treatments (S2 + AC and S1 + AC) resulted in significantly lower progeny for all insect species compared to single treatments (Table 8). Although no suppressed progeny was observed, the minimum progeny production was demonstrated for S2 + AC, followed by S1 + AC, S2, S1, and AC among all insect species and storage periods. The overall offspring production demonstrated a decline with time for all species tested. *Rhyzopertha dominica* demonstrated minimum progeny among all insect species regardless of the treatment and storage period, followed by *S. oryzae*, *T. castaneum*, *C. ferrugineus*, *T. granarium*, and *O. surinamensis.*

## 4. Discussion

In this study, the single and combined effects of spinosad and alpha-cypermethrin were investigated as wheat protectants against six distinct storage pests in laboratory and persistence bioassays. In single applications, both concentrations of spinosad induced higher mortality than alpha-cypermethrin alone, irrespective of the dose. However, the combined treatments exhibited significantly higher mortalities when compared to the application of each component alone. Among the various stored-grain pests and treatments assessed, the highest susceptibility was observed for *R. dominica* followed by *S. oryzae*, *T. castaneum*, *C. ferrugineus*, *O. surinamensis*, and *T. granarium*.

In the present study, alpha-cypermethrin demonstrated high efficacy in controlling stored-grain pests. Previously, the efficacy of alpha-cypermethrin and thiamethoxam on concrete against larvae of *T. granarium* and *T. molitor* has been explored, and alpha-cypermethrin was proven more efficient than thiamethoxam [32]. Furthermore, larvae previously exposed to alpha-cypermethrin exhibited higher delayed mortality compared to those exposed to thiamethoxam. Additionally, while the immediate mortalities of *S. oryzae* and *R. dominica* did not significantly differ from controls when treated with alpha-cypermethrin on polypropylene bags, delayed mortality was notably high [33]. In addition, alpha-cypermethrin at a high rate (0.250 mg/kg) showed significant effectiveness in controlling *T. confusum* on stored wheat, with mortality rates of 87.4% at 1 month and 55.3% at 3.5 months after treatment, while it also disrupted the reproductive capabilities of the surviving insects [31].

In both the laboratory and persistence bioassays conducted in this study, spinosad provided more effective control of the pest species tested than alpha-cypermethrin. Spinosad has been extensively investigated for the management of storage pests in the past. Vayias et al. [21] reported that the impact of spinosad on *S. oryzae* varied depending on the wheat variety, resulting in mortality rates ranging from 29.3% to 53.3% at 7 days of exposure. These findings align closely with the results of the present study, which indicated a mortality rate of 39.63% in *S. oryzae* at 7 days post-exposure. Previously, a study by Kavallieratos et al. [46] investigated the susceptibility of *T. granarium* to spinosad at a concentration of 0.5 mg/kg wheat. In their research, they reported significantly high adult mortality rates of 93.3% and 97.8% at 7- and 14-days post-exposure on wheat. In the current study, a dose of 0.1 mg/kg wheat resulted to lower mortality rates of 22.39% and 37.21% at the same exposure intervals. Discrepancies in the results may be attributed to variations in the dose administered. Furthermore, in an assessment of the efficacy of spinosad against susceptible and tolerant strains of various storage pests on wheat, significantly higher mortalities were reported than those observed in this study [18]. Specifically, 99.3% and 100% mortality were reported for *R. dominica* at 7- and 14-days post-exposure at a dose of 0.1 mg/kg wheat, compared to the lower mortalities of 41.3% and 59.05% observed in our study [18]. Additionally, very low mortality rates of 0.6% and 0% were reported for *S. oryzae* and *T. castaneum*, respectively [18], for both exposures at the same dose, contrasting with our study’s findings of 39.63–56.97% mortality for *S. oryzae* and 35.58–51.19% mortality for *T. castaneum* during 7–14 days of exposure. The significance of *T. castaneum* exhibiting elevated mortality lies in the fact that, owing to the distinct characteristics of insects, various pesticide susceptibility and tolerance patterns emerge for each insect species, developmental stage, or population [38,47]. Previously, the application of 1 ppm of spinosad did not achieve comprehensive control over this pest, while it mostly eliminated progeny production [48]. As exhibited in the aforementioned literature, insecticide efficacy is influenced by the pest species or strain, the dose administered, and the type of substrate used, highlighting the need for tailored and context-specific pest management strategies to optimize control outcomes.

Various insecticide combinations can yield diverse outcomes in pest mortality, underscoring the need for comprehensive exploration and optimization of these combinations. For instance, while the mixture of lambda-cyhalothrin, clove oil, and alpha-cypermethrin has been reported to significantly enhance efficacy against *R. dominica*, combining alpha-cypermethrin with malathion resulted in reduced effectiveness [34]. Furthermore, combining diatomaceous earths (DEs) with chemical pesticides [49], plant extracts [50], or entomopathogenic fungi [51,52] has been proven to enhance efficacy against storage pests compared to single applications of each insecticidal component. Islam et al. [53], who conducted a study examining the effects of insecticides based on DE (protect-It and silicoSec), as well as the nano-structured silica product AL06, along with monoterpenoids (eugenol and cinnamaldehyde) on *Callosobruchus maculatus* (F.) (Coleoptera: Chrysomelidae) and *S. oryzae*, revealed predominantly antagonistic interactions in experiments with *C. maculatus*, while synergistic interactions were specifically observed in combinations involving SilicoSec combined with cinnamaldehyde and eugenol against *S. oryzae*. In the current study, the joint action of spinosad and alpha-cypermethrin exhibited significantly enhanced efficacy against all stored-grain pests tested in both laboratory and persistence bioassays compared to each insecticidal component alone. Previous research has demonstrated an enhanced effect resulting from the combination of spinosad with pyrethrins. The joint action of spinosad with fenpropathrin against larvae of *Spodoptera littoralis* (Boisduval) (Lepidoptera: Noctuidae) demonstrated a synergistic effect, as indicated by co-toxicity factors ranging from +31.37 to +47.54 [12]. This potentiation effect suggests that the combination of fenpropathrin and spinosad can significantly enhance control efficacy, possibly allowing for a reduction in the doses of one or both constituents of the combination [12]. In the current research, combined treatments resulted in significantly higher mortalities compared to single treatments for all insect species. Nevertheless, the joint treatments did not always yield significant differences from one another, highlighting the fact that this insecticidal combination may be used at the lower dose providing similar results. Application rate reduction aims to mitigate the issue of resistance emergence in storage pests, particularly against organophosphates and pyrethroids, which have been overused for pest control [7]. To combat this problem, strategies involve diminishing the inordinate application of these insecticides and considering alternative insecticide groups with different modes of action [54,55]. Pertaining to this, spinosad enhanced the susceptibility of insecticide-resistant *Alphitobius diaperinus* Panzer (Coleoptera: Tenebrionidae) populations to pyrethroids, while non-resistant populations showed no susceptibility changes [56]. The fact that the combination of spinosad and a pyrethroid, such as alpha-cypermethrin, resulted to enhanced mortalities to all tested species might indicate that synergism is expanded beyond resistant populations. However, this important finding merits further investigation. The results of the current study yield elevated mortalities concerning the combined treatments, even in pests known to have tolerance in conventional insecticides, such as *T. castaneum*, *T. granarium*, *R. dominica*, and *O. surinamensis* [38,57,58], while the elevated mortalities in persistence trials, even at 120 days after exposure to the combined treatments, underscores the potential suitability of this combination for extended storage periods in both packaged and unpackaged stored commodities.

The efficacy of an insecticidal component as a management tool was effectively demonstrated through both the mortality rates and reduction in offspring for the treated groups in comparison to the control group. Progeny suppression was particularly pronounced among the species that exhibited high mortalities. While complete suppression of progeny was not attained, it consistently remained significantly lower than the control group for all insect species, in both single and combined treatments, while the combined treatments exhibited significantly lower progeny compared to the single ones. This trend was observed in both laboratory and persistence trials. This was evident in the case of *R. dominica* when the S2 + AC combination was used. The partial survival of parental adults might account for the persistence of progeny production. In a prior study, the application of spinosad at 0.1 and 0.5 ppm did not completely inhibit the emergence of offspring in *S. oryzae*, *Sitophilus granarius* (L.) (Coleoptera: Curculionidae), and *Liposcelis bostrychophila* Badonnel (Psocoptera: Liposcelidae) [38]. It is worth noting that the absolute suppression of progeny is not always achieved, even in cases where 100% adult mortality is attained [38,59].

## 5. Conclusions

The present study highlights the remarkable effectiveness of combining spinosad and alpha-cypermethrin against storage pests. While individual treatments with either insecticide were effective in reducing pest populations of *R. dominica*, *S. oryzae*, *T. granarium*, *O. surinamensis*, *C. ferrugineus*, and *T. castaneum*, the combined treatment exhibited significantly higher mortality rates and concurrently reduced progeny production. Notably, this study marks the first examination of the combined action of spinosad and alpha-cypermethrin, revealing its promising enhanced effect. This innovative approach not only enhances pest control efficacy but also offers the potential to decrease the required dose of one or both elements within the blend. However, further research encompassing different commodities, temperatures, species, and doses is essential to refine and validate this approach as a comprehensive and reliable management tool for diverse storage pest scenarios.

## Figures and Tables

**Table 1:** ANOVA parameters for adult mortality of *R. dominica*, *S. oryzae*, *T. castaneum*, *C. ferrugineus*, *O. surinamensis*, and *T. granarium* on wheat treated with two doses of spinosad, one dose of alpha-cypermethrin, and their respective combinations in laboratory bioassays (total df = 89).

Effect	df	*R. dominica*		*S. oryzae*		*T. castaneum*		*C. ferrugineus*		*O. surinamensis*		*T. granarium*	
		F	*p*	F	*p*	F	*p*	F	*p*	F	*p*	F	*p*
Treatment	4	57.34	<0.01	51.86	<0.01	172.10	<0.01	202.71	<0.01	155.15	<0.01	143.38	<0.01
Exposure	2	80.98	<0.01	104.61	<0.01	348.29	<0.01	405.41	<0.01	292.79	<0.01	270.06	<0.01
Treatment × exposure	8	19.27	<0.01	3.63	<0.01	14.02	<0.01	18.14	<0.01	12.67	<0.01	11.52	<0.01

**Table 2:** Mean mortality (% ± SE) of *R. dominica*, *S. oryzae*, *T. castaneum*, *C. ferrugineus*, *O. surinamensis*, and *T. granarium* adults after a 3-, 7- and 14-day exposure on wheat treated with two doses of spinosad at 0.05 mg/kg (S1) and 0.1 mg/kg (S2), one dose of alpha-cypermethrin at 0.05 mg/kg (AC), and their respective combinations, S1 + AC and S2 + AC, in laboratory bioassays. For each species, within each row, means followed by the same uppercase letter are not significantly different (Tukey–Kramer (HSD) test at *p* = 0.05). For each species, within each column, means followed by the same lowercase letter are not significantly different (Tukey–Kramer (HSD) test at *p* = 0.05).

Species	Treatment	Days Post-Exposure			F_2, 17_	*p*
		3	7	14		
*R. dominica*	S1	18.41 ± 4.69 Ccd	33.55 ± 4.38 Bbc	47.15 ± 3.55 Ac	11.4	<0.01
	S2	29.22 ± 2.59 Bbc	41.30 ± 5.22 Bbc	59.05 ± 1.51 Ab	18.6	<0.01
	AC	14.40 ± 2.47 Cd	26.08 ± 2.77 Bc	38.57 ± 3.92 Ac	15.0	<0.01
	S1 + AC	36.27 ± 2.26 Cab	49.53 ± 5.04 Bab	87.00 ± 2.55 Aa	56.0	<0.01
	S2 + AC	43.32 ± 2.3 Ca	66.10 ± 2.12 Ba	98.29 ± 0.62 Aa	218	<0.01
	F_4, 29_	15.9	14.2	88.5		
	*p*	<0.01	<0.01	<0.01		
*S. oryzae*	S1	16.67 ± 1.38 Bab	30.51 ± 3.34 ABcd	44.38 ± 1.74 Ac	3.44	0.05
	S2	26.88 ± 2.60 Cab	39.63 ± 3.67 Bbc	56.97 ± 1.94 Ab	28.4	<0.01
	AC	12.72 ± 2.64 Cb	24.72 ± 2.21 Bd	35.84 ± 3.29 Ac	17.6	<0.01
	S1 + AC	34.23 ± 1.67 Cab	45.44 ± 3.83 Bb	84.62 ± 2.06 Aa	96.4	<0.01
	S2 + AC	40.27 ± 1.32 Ca	62.36 ± 2.41 Ba	93.87 ± 2.29 Aa	169	<0.01
	F_4, 29_	3.90	21.2	117		
	*p*	<0.01	<0.01	<0.01		
*T. castaneum*	S1	14.12 ± 3.97 Ccd	27.11 ± 2.20 Bcd	40.60 ± 1.56 Ad	22.8	<0.01
	S2	23.85 ± 1.81 Cbc	35.58 ± 1.51 Bbc	51.19 ± 1.45 Ac	73.2	<0.01
	AC	10.04 ± 1.44 Cd	21.34 ± 1.70 Bd	32.74 ± 1.76 Ad	47.8	<0.01
	S1 + AC	31.19 ± 1.87 Bab	40.36 ± 3.43 Bb	81.56 ± 2.36 Ab	103	<0.01
	S2 + AC	37.22 ± 2.04 Ca	56.59 ± 1.24 Ba	89.75 ± 2.36 Aa	187	<0.01
	F_4, 29_	22.4	39.4	168		
	*p*	<0.01	<0.01	<0.01		
*C. ferrugineus*	S1	11.42 ± 3.46 Cbc	24.05 ± 1.73 Bcd	36.18 ± 1.65 Ac	25.9	<0.01
	S2	19.12 ± 0.87 Cb	31.54 ± 1.61 Bbc	46.76 ± 1.58 Ab	97.3	<0.01
	AC	7.36 ± 1.09 Cc	18.62 ± 2.02 Bd	27.30 ± 2.50 Ad	25.9	<0.01
	S1 + AC	27.17 ± 1.29 Ca	36.27 ± 2.09 Bb	77.45 ± 1.24 Aa	283	<0.01
	S2 + AC	33.53 ± 1.74 Ca	51.51 ± 2.22 Ba	82.24 ± 2.15 Aa	144	<0.01
	F_4, 29_	31.2	42.0	171		
	*p*	<0.01	<0.01	<0.01		
*O. surinamensis*	S1	9.41 ± 2.42 Cc	21.36 ± 1.29 Bcd	32.38 ± 2.36 Abc	30.1	<0.01
	S2	16.76 ± 0.95 Cb	27.13 ± 2.53 Bbc	41.66 ± 2.11 Ab	39.7	<0.01
	AC	5.68 ± 149 Cc	15.24 ± 1.44 Bd	23.56 ± 2.84 Ac	19.3	<0.01
	S1 + AC	24.17 ± 0.54 Ca	32.87 ± 1.41 Bb	67.56 ± 3.04 Aa	136	<0.01
	S2 + AC	29.52 ± 1.74 Ca	47.15 ± 2.58 Ba	76.80 ± 2.31 Aa	114	<0.01
	F_4, 29_	39.7	39.4	79.9		
	*p*	<0.01	<0.01	<0.01		
*T. granarium*	S1	7.72 ± 2.40 Cbc	18.64 ± 1.43 Bcd	29.66 ± 2.51 Ab	25.5	<0.01
	S2	13.07 ± 1.51 Cb	22.39 ± 2.81 Bbc	37.21 ± 1.68 Ab	34.1	<0.01
	AC	4.68 ± 1.51 Cc	13.20 ± 1.77 Bd	20.50 ± 1.73 Ac	22.2	<0.01
	S1 + AC	20.76 ± 1.77 Ca	29.81 ± 2.03 Bb	62.11 ± 1.71 Aa	138	<0.01
	S2 + AC	26.16 ± 1.83 Ca	44.42 ± 2.66 Ba	70.31 ± 2.25 Aa	94.9	<0.01
	F_4, 29_	23.7	30.0	113		
	*p*	<0.01	<0.01	<0.01		

**Table 3:** ANOVA parameters for progeny production *R. dominica*, *S. oryzae*, *T. castaneum*, *C. ferrugineus*, *O. surinamensis*, and *T. granarium* on wheat treated with spinosad and alpha-cypermethrin in laboratory bioassays (total df = 215).

Effect	df	F	*p*
Treatment	5	808.89	<0.01
Species	5	226.69	<0.01
Treatment × species	25	4.16	<0.01

**Table 4:** Mean progeny number (±SE) of *R. dominica*, *S. oryzae*, *T. castaneum*, *C. ferrugineus*, *T. granarium*, and *O. surinamensis* individuals/vial following a 62, 60-, 62-, 65-, 46-, and 65-day exposure interval, respectively, on wheat treated with two doses of spinosad at 0.05 mg/kg (S1) and 0.1 mg/kg (S2), one dose of alpha-cypermethrin at 0.05 mg/kg (AC), and their respective combinations, S1 + AC and S2 + AC, in laboratory bioassays. For each species, within each column, means followed by the same lowercase letter are not significantly different (Tukey–Kramer (HSD) test at *p* = 0.05).

Treatment	*R. dominica*	*S. oryzae*	*T. castaneum*	*C. ferrugineus*	*O. surinamensis*	*T. granarium*
S1	56.95 ± 2.85 bc	63.98 ± 3.81 bc	75.98 ± 2.89 bc	86.53 ± 2.50 bc	115.68 ± 3.11 b	98.06 ± 3.13 bc
S2	46.00 ± 3.15 c	51.26 ± 2.69 c	66.58 ± 3.09 c	78.61 ± 3.08 c	94.85 ± 3.07 c	86.61 ± 3.02 c
AC	67.11 ± 2.98 b	74.91 ± 3.18 b	87.55 ± 2.91 b	99.66 ± 3.00 b	128.82 ± 2.77 b	110.03 ± 3.11 b
S1 + AC	19.53 ± 2.29 d	24.76 ± 3.14 d	31.51 ± 3.06 d	37.73 ± 2.40 d	65.93 ± 3.08 d	45.75 ± 3.02 d
S2 + AC	7.98 ± 1.77 d	15.96 ± 3.23 d	24.80 ± 3.06 d	31.50 ± 3.60 d	40.10 ± 3.20 e	58.05 ± 3.23 d
Control	112.18 ± 3.02 a	102.70 ± 3.07 a	119.43 ± 2.32 a	119.58 ± 3.65 a	151.75 ± 3.05 a	132.93 ± 3.14 a
F_5, 35_	172.0	101.0	149.0	127.0	183.0	109.0
*p*	<0.01	<0.01	<0.01	<0.01	<0.01	<0.01

**Table 5:** ANOVA parameters for adult mortality of *R. dominica*, *S. oryzae*, *T. castaneum*, *C. ferrugineus*, *O. surinamensis*, and *T. granarium* on wheat treated with two doses of spinosad, one dose of alpha-cypermethrin, and their respective combinations in persistence bioassays (total df = 149).

Effect	df	*R. dominica*		*S. oryzae*		*T. castaneum*		*C. ferrugineus*		*O. surinamensis*		*T. granarium*	
		F	*p*	F	*p*	F	*p*	F	*p*	F	*p*	F	*p*
Treatment	4	279.63	<0.01	421.12	<0.01	619.29	<0.01	795.03	<0.01	593.25	<0.01	556.99	<0.01
Storage period	4	42.34	<0.01	72.46	<0.01	110.55	<0.01	130.56	<0.01	94.38	<0.01	88.90	<0.01
Treatment × storage period	16	0.42	0.97	0.93	0.54	1.41	0.14	2.01	0.017	2.56	<0.01	2.73	<0.01

**Table 6:** Mean mortality (% ± SE) of *R. dominica*, *S. oryzae*, *T. castaneum*, *C. ferrugineus*, *O. surinamensis*, and *T. granarium* adults after a 7-day exposure on wheat treated with two doses of spinosad at 0.05 mg/kg (S1) and 0.1 mg/kg (S2), one dose of alpha-cypermethrin at 0.05 mg/kg (AC), and their respective combinations, S1 + AC and S2 + AC, in five storage periods carried out from 0 to 120 days after treatment. Within each row, means followed by the same uppercase letter are not significantly different (Tukey–Kramer (HSD) test at *p* = 0.05). For each species, within each column, means followed by the same lowercase letter are not significantly different (Tukey–Kramer (HSD) test at *p* = 0.05).

Species	Treatment	Storage Period					F_4, 29_	*p*
		0 Days	30 Days	60 Days	90 Days	120 Days		
*R. dominica*	S1	42.26 ± 8.05 Ab	35.12 ± 2.85 ABbc	31.38 ± 2.41 ABcd	26.62 ± 1.49 ABd	20.48 ± 1.08 Bd	4.16	<0.01
	S2	53.54 ± 4.79 Ab	47.39 ± 4.18 ABb	42.29 ± 4.15 ABCc	37.52 ± 2.23 BCc	31.37 ± 1.95 Cc	5.53	<0.01
	AC	34.42 ± 5.10 Ab	29.30 ± 4.45 ABc	23.51 ± 2.36 ABCd	18.07 ± 1.4 BCe	14.32 ± 1.27 Cd	6.02	<0.01
	S1 + AC	82.91 ± 3.77 Aa	76.45 ± 3.14 ABa	67.26 ± 3.68 BCb	58.72 ± 2.25 CDb	50.85 ± 1.50 Db	18.6	<0.01
	S2 + AC	94.52 ± 1.80 Aa	89.42 ± 2.54 ABa	82.27 ± 2.06 BCa	76.09 ± 1.52 CDa	69.28 ± 2.51 Da	22.5	<0.01
	F_4, 29_	25.8	55.7	65.7	170	169		
	*p*	<0.01	<0.01	<0.01	<0.01	<0.01		
*S. oryzae*	S1	39.54 ± 4.11 Abc	31.38 ± 2.36 ABbc	27.64 ± 1.25 BCc	23.20 ± 2.06 BCd	17.75 ± 1.14 Cd	11.5	<0.01
	S2	50.48 ± 5.04 Ab	43.65 ± 4.76 ABb	36.50 ± 3.02 ABCd	32.39 ± 2.26 BCc	27.62 ± 2.53 Cc	5.96	<0.01
	AC	31.02 ± 3.08 Ac	25.56 ± 4.62 ABc	19.77 ± 1.32 BCd	15.69 ± 0.84 BCe	10.58 ± 1.44 Ce	9.10	<0.01
	S1 + AC	78.52 ± 1.91 Aa	71.32 ± 2.13 ABa	63.17 ± 1.66 Bb	52.20 ± 1.63 Cb	46.08 ± 1.45 Cb	56.4	<0.01
	S2 + AC	87.04 ± 1.44 Aa	82.60 ± 1.12 ABa	76.46 ± 1.78 BCa	70.30 ± 1.87 Ca	61.76 ± 1.42 Da	41.5	<0.01
	F_4, 29_	51.7	56.3	161	153	157		
	*p*	<0.01	<0.01	<0.01	<0.01	<0.01		
*T. castaneum*	S1	36.82 ± 2.72 Abc	27.31 ± 1.04 Bd	24.22 ± 1.90 BCd	18.42 ± 1.47 CDd	13.64 ± 1.11 Dd	25.2	<0.01
	S2	46.40 ± 2.21 Ab	39.22 ± 1.33 ABc	32.39 ± 2.26 BCc	29.32 ± 2.22 BCc	21.50 ± 1.27 Cc	10.1	<0.01
	AC	27.96 ± 2.17 Ac	21.15 ± 1.33 Be	16.71 ± 1.08 BCe	12.63 ± 0.84 CDd	8.20 ± 1.20 Dd	26.9	<0.01
	S1 + AC	72.01 ± 2.10 Aa	67.58 ± 1.41 Ab	57.33 ± 1.35 Bb	48.46 ± 1.85 Cb	41.29 ± 1.97 Cb	51.2	<0.01
	S2 + AC	80.20 ± 1.46 Aa	78.49 ± 1.41 Aa	70.30 ± 1.87 Ba	65.85 ± 2.72 Ba	57.68 ± 1.97 Ca	26.1	<0.01
	F_4, 29_	50.4	382	169	130	211		
	*p*	<0.01	<0.01	<0.01	<0.01	<0.01		
*C. ferrugineus*	S1	31.36 ± 2.81 Ac	23.20 ± 1.22 ABd	19.43 ± 2.99 BCcd	14.34 ± 1.83 BCd	10.21 ± 1.73 Cd	13.4	<0.01
	S2	40.59 ± 1.23 Ab	35.14 ± 2.17 Ac	27.28 ± 1.58 Bc	26.26 ± 2.09 Bc	18.09 ± 0.63 Cc	27.7	<0.01
	AC	23.53 ± 1.21 Ac	18.76 ± 0.94 Bd	13.31 ± 1.27 Cd	10.24 ± 1.05 CDd	6.48 ± 0.97 Dd	38.0	<0.01
	S1 + AC	66.57 ± 2.15 Aa	63.47 ± 1.28 Ab	52.53 ± 17 Bb	45.40 ± 1.28 Cb	37.55 ± 1.05 Db	53.5	<0.01
	S2 + AC	75.09 ± 2.63 Aa	74.38 ± 1.45 ABa	66.56 ± 1.39 BCa	60.73 ± 1.69 Ca	52.53 ± 2.17 Da	24.4	<0.01
	F_4, 29_	111	281	131	170	186		
	*p*	<0.01	<0.01	<0.01	<0.01	<0.01		
*O. surinamensis*	S1	28.64 ± 2.30 Ab	19.79 ± 1.02 Bd	16.36 ± 1.46 BCcd	12.64 ± 1.46 CDd	8.51 ± 1.32 Dcd	23.6	<0.01
	S2	36.17 ± 2.44 Ab	31.40 ± 0.73 ABc	24.56 ± 2.39 Bc	26.26 ± 2.09 Bc	14.64 ± 2.18 Cc	15.3	<0.01
	AC	18.43 ± 1.58 Ac	14.68 ± 1.24 ABd	10.22 ± 1.38 BCd	10.24 ± 1.05 BCd	5.12 ± 0.87 Cd	16.2	<0.01
	S1 + AC	61.40 ± 2.07 Aa	55.30 ± 1.85 ABb	48.09 ± 2.98 BCb	41.28 ± 2.01 Cb	32.07 ± 1.93 Db	27.3	<0.01
	S2 + AC	69.26 ± 2.98 Aa	67.62 ± 1.26 Aa	61.12 ± 2.55 ABa	55.29 ± 1.07 BCa	46.75 ± 1.50 Ca	19.6	<0.01
	F_4, 29_	87.6	237	93.3	143	116		
	*p*	<0.01	<0.01	<0.01	<0.01	<0.01		
*T. granarium*	S1	22.86 ± 1.59 Ad	16.05 ± 1.81 ABd	12.28 ± 1.65 BCd	9.89 ± 2.06 BCd	6.46 ± 2.26 Cd	11.0	<0.01
	S2	31.75 ± 1.21 Ac	27.31 ± 1.16 ABc	20.81 ± 0.62 BCc	15.68 ± 3.17 CDc	11.24 ± 1.71 Dc	21.4	<0.01
	AC	15.70 ± 1.55 Ad	10.23 ± 1.74 ABd	8.18 ± 1.89 BCd	5.81 ± 1.54 BCd	3.08 ± 0.88 Cd	9.34	<0.01
	S1 + AC	56.30 ± 1.97 Ab	51.19 ± 2.53 ABb	44.69 ± 2.19 BCb	36.18 ± 2.28 Cb	27.31 ± 1.41 Db	30.1	<0.01
	S2 + AC	63.81 ± 2.16 Aa	62.42 ± 2.38 Aa	57.67 ± 1.73 ABa	51.16 ± 2.37 Ba	40.60 ± 1.26 Ca	21.9	<0.01
	F_4, 29_	147	129	158	67.1	100		
	*p*	<0.01	<0.01	<0.01	<0.01	<0.01		

**Table 7:** ANOVA parameters for progeny production *R. dominica*, *S. oryzae*, *T. castaneum*, *C. ferrugineus*, *O. surinamensis*, and *T. granarium* on wheat treated with spinosad and alpha-cypermethrin in persistence bioassays (total df = 179).

Effect	df	*R. dominica*		*S. oryzae*		*T. castaneum*		*C. ferrugineus*		*O. surinamensis*		*T. granarium*	
		F	*p*	F	*p*	F	*p*	F	*p*	F	*p*	F	*p*
Treatment	5	1.37	0.23	2.12	0.06	2.19	0.05	2.39	0.04	1.25	0.29	2.43	0.03
Storage period	4	3.98	<0.01	5.10	<0.01	4.68	<0.01	5.42	<0.01	1.46	0.21	5.88	<0.01
Treatment × storage period	20	0.05	1.00	0.00	1.00	0.00	1.00	0.00	1.00	0.00	1.00	0.00	1.00

**Table 8:** Mean progeny number (±SE) of *R. dominica*, *S. oryzae*, *T. castaneum*, *C. ferrugineus*, *O. surinamensis*, and *T. granarium* individuals/vial on wheat treated with two doses of spinosad at 0.05 mg/kg (S1) and 0.1 mg/kg (S2), one dose of alpha-cypermethrin at 0.05 mg/kg (AC), and their respective combinations, S1 + AC and S2 + AC, in five storage periods carried out from 0 to 120 days after treatment. Within each row, means followed by the same uppercase letter are not significantly different (Tukey–Kramer (HSD) test at *p* = 0.05). For each species, within each column, means followed by the same lowercase letter are not significantly different (Tukey–Kramer (HSD) test at *p* = 0.05).

Species	Treatment	Storage Period					F_4, 29_	*p*
		0 Days	30 Days	60 Days	90 Days	120 Days		
*R. dominica*	S1	67.33 ± 3.07 Cbc	74.76 ± 3.03 BCbc	86.70 ± 3.05 ABbc	94.38 ± 3.67 Abc	99.33 ± 3.59 Abc	16.2	<0.01
	S2	58.33 ± 2.36 Cc	63.38 ± 2.84 BCc	75.83 ± 3.06 ABc	81.41 ± 3.65 Ac	87.46 ± 3.64 Ac	14.5	<0.01
	AC	74.33 ± 3.07 Cb	85.81 ± 3.04 BCb	97.21 ± 3.18 ABb	105.48 ± 3.63 ABb	110.47 ± 6.63 Ab	19.3	<0.01
	S1 + AC	29.34 ± 3.00 Cd	38.90 ± 3.05 BCd	50.15 ± 3.06 ABd	49.50 ± 3.59 ABd	57.63 ± 3.69 Ad	10.9	<0.01
	S2 + AC	16.34 ± 3.08 Ce	27.53 ± 2.83 BCd	38.15 ± 3.24 ABd	43.55 ± 3.71 ABd	49.48 ± 3.65 Ad	15.2	<0.01
	Control	119.00 ± 2.92 Ba	123.87 ± 3.09 ABa	129.63 ± 3.70 ABa	130.72 ± 3.61 ABa	136.60 ± 3.61 Aa	3.88	<0.01
	F_4, 29_	154	134	106	83.9	81.1		
	*p*	<0.01	<0.01	<0.01	<0.01	<0.01		
*S. oryzae*	S1	71.45 ± 3.60 Cb	79.46 ± 3.61 BCbc	91.43 ± 3.65 ABbc	99.50 ± 3.69 Abc	106.55 ± 3.70 Abc	15.4	<0.01
	S2	65.45 ± 3.72 Bb	68.60 ± 3.60 Bc	79.50 ± 3.58 ABc	85.43 ± 3.66 Ac	92.58 ± 3.67 Ac	9.67	<0.01
	AC	79.33 ± 3.63 Cb	91.58 ± 3.63 BCb	102.67 ± 3.63 ABb	109.47 ± 3.61 Ab	117.53 ± 3.65 Ab	17.0	<0.01
	S1 + AC	34.51 ± 3.62 Cc	43.43 ± 3.62 BCd	56.63 ± 3.74 ABd	62.58 ± 3.60 Ad	69.43 ± 3.61 Ad	15.2	<0.01
	S2 + AC	21.60 ± 3.68 Cc	32.65 ± 3.65 BCd	41.75 ± 3.64 ABd	49.60 ± 3.62 Ad	56.56 ± 3.64 Ad	14.3	<0.01
	Control	123.62 ± 3.64 Ba	127.53 ± 3.63 ABa	132.63 ± 3.65 ABa	134.70 ± 3.70 ABa	139.53 ± 3.63 Aa	2.88	<0.01
	F_4, 29_	97.2	89.3	79.7	73.0	70.9		
	*p*	<0.01	<0.01	<0.01	<0.01	<0.01		
*T. castaneum*	S1	78.35 ± 3.65 Db	85.35 ± 3.67 CDbc	97.48 ± 3.65 BCbc	105.35 ± 3.68 ABbc	113.63 ± 3.64 Abc	15.4	<0.01
	S2	72.53 ± 3.68 Cb	77.20 ± 3.66 BCc	82.40 ± 3.64 BCc	91.35 ± 3.64 ABc	98.46 ± 3.65 Ac	8.27	<0.01
	AC	85.38 ± 3.65 Cb	94.50 ± 3.68 BCb	106.60 ± 3.67 ABb	114.42 ± 3.68 Ab	121.60 ± 3.58 Ab	16.1	<0.01
	S1 + AC	39.38 ± 3.55 Dc	49.31 ± 3.57 CDd	59.41 ± 3.61 BCd	67.36 ± 3.70 ABd	74.66 ± 3.67 Ad	15.0	<0.01
	S2 + AC	28.31 ± 3.62 Cc	35.55 ± ±3.64 BCd	45.58 ± 3.67 ABd	54.50 ± 3.74 Ad	59.48 ± 3.61 Ad	12.1	<0.01
	Control	128.50 ± 3.65 Aa	132.68 ± 3.65 Aa	137.48 ± 3.59 Aa	139.50 ± 3.60 Aa	141.65 ± 3.57 Aa	2.17	<0.01
	F_4, 29_	96.5	89.0	83.3	72.1	70.8		
	*p*	<0.01	<0.01	<0.01	<0.01	<0.01		
*C. ferrugineus*	S1	85.36 ± 3.67 Cb	89.48 ± 3.66 BCbc	104.47 ± 3.70 ABb	111.48 ± 3.62 Ab	119.62 ± 3.65 Ab	15.6	<0.01
	S2	79.43 ± 3.67 Cb	81.41 ± 3.67 BCc	87.43 ± 3.68 BCc	95.48 ± 3.67 ABc	103.47 ± 3.66 Ac	7.45	<0.01
	AC	94.45 ± 3.69 Cb	99.40 ± 3.66 BCb	112.63 ± 3.67 ABb	121.60 ± 3.61 Ab	127.60 ± 3.66 Ab	14.9	<0.01
	S1 + AC	47.66 ± 3.67 Dc	54.50 ± 3.62 CDd	65.40 ± 3.65 BCd	72.60 ± 3.64 ABd	82.40 ± 3.62 Ad	14.5	<0.01
	S2 + AC	34.56 ± 3.65 Dc	39.46 ± 3.62 CDd	51.40 ± 3.67 BCd	62.73 ± 3.64 ABd	71.28 ± 3.60 Ad	17.9	<0.01
	Control	129.57 ± 3.62 Ba	134.55 ± 3.66 ABa	139.40 ± 3.62 ABa	141.60 ± 3.65 ABa	145.57 ± 3.63 Aa	2.93	<0.01
	F_4, 29_	86.2	85.1	77.1	67.6	59.6		
	*p*	<0.01	<0.01	<0.01	<0.01	<0.01		
*O. surinamensis*	S1	119.03 ± 3.77 BCb	107.45 ± 3.62 Cc	119.38 ± 3.69 BCc	127.38 ± 3.66 ABc	141.53 ± 3.60 Ab	11.7	<0.01
	S2	99.45 ± ±3.67 ABc	92.50 ± 3.63 Bc	98.55 ± 3.64 ABb	103.30 ± 3.62 ABb	110.60 ± 3.64 Ac	3.35	<0.01
	AC	131.47 ± 47 Bb	125.68 ± 3.62 Bb	132.52 ± 3.60 Bb	139.30 ± 3.60 ABb	151.35 ± 3.61 Ab	7.37	<0.01
	S1 + AC	69.40 ± 3.61 ABd	61.43 ± 3.65 Bd	68.35 ± 3.58 ABd	74.33 ± 3.63 ABd	82.51 ± 3.69 Ad	4.63	<0.01
	S2 + AC	45.51 ± 3.70 ABe	36.65 ± 3.66 Be	45.35 ± 3.67 ABe	53.43 ± 3.58 Ae	59.48 ± 3.59 Ae	5.71	<0.01
	Control	159.57 ± 3.61 Ba	161.43 ± ±3.64 Ba	165.37 ± 3.68 ABa	171.38 ± 3.60 ABa	179.47 ± 3.62 Aa	4.97	<0.01
	F_4, 29_	129	151	143	144	154		
	*p*	<0.01	<0.01	<0.01	<0.01	<0.01		
*T. granarium*	S1	91.50 ± 3.56 Db	95.50 ± 3.72 CDc	110.60 ± 3.63 BCb	119.53 ± 3.65 ABb	130.52 ± 3.59 Ab	20.1	<0.01
	S2	85.51 ± 3.71 Bb	87.36 ± 3.64 Bc	92.56 ± 3.66 Bc	99.46 ± 3.67 ABc	110.57 ± 3.63 Ac	7.75	<0.01
	AC	99.58 ± 3.65 Db	104.45 ± 3.63 CDb	116.58 ± 3.68 BCb	125.45 ± 3.68 ABb	132.75 ± 3.62 Ab	14.4	<0.01
	S1 + AC	52.56 ± 3.68 Dc	57.50 ± 3.64 CDd	69.51 ± 3.66 BCd	76.38 ± ±3.63 ABd	87.30 ± 3.66 Ad	14.8	<0.01
	S2 + AC	39.56 ± 3.61 Cc	45.46 ± 3.71 Cd	54.45 ± 3.70 BCd	68.36 ± 3.56 ABd	76.43 ± 3.62 Ad	17.9	<0.01
	Control	131.62 ± 3.62 Ba	137.48 ± 3.53 ABa	141.38 ± 3.62 ABa	145.53 ± 3.61 ABa	148.52 ± 3.61 Aa	3.42	<0.01
	F_4, 29_	83.4	82.4	76.3	67.4	60.1		
	*p*	<0.01	<0.01	<0.01	<0.01	<0.01		

## Data Availability

Data are available within the article.
